# A deep learning adversarial autoencoder with dynamic batching displays high performance in denoising and ordering scRNA-seq data

**DOI:** 10.1016/j.isci.2024.109027

**Published:** 2024-01-30

**Authors:** Kyung Dae Ko, Vittorio Sartorelli

**Affiliations:** 1 Laboratory of Muscle Stem Cells & Gene Regulation, NIAMS, NIH, Bethesda, MD, USA

**Keywords:** Natural sciences, Biological sciences, Molecular biology, Bioinformatics, Sequence analysis, Omics

## Abstract

By providing high-resolution of cell-to-cell variation in gene expression, single-cell RNA sequencing (scRNA-seq) offers insights into cell heterogeneity, differentiating dynamics, and disease mechanisms. However, challenges such as low capture rates and dropout events can introduce noise in data analysis. Here, we propose a deep neural generative framework, the dynamic batching adversarial autoencoder (DB-AAE), which excels at denoising scRNA-seq datasets. DB-AAE directly captures optimal features from input data and enhances feature preservation, including cell type-specific gene expression patterns. Comprehensive evaluation on simulated and real datasets demonstrates that DB-AAE outperforms other methods in denoising accuracy and biological signal preservation. It also improves the accuracy of other algorithms in establishing pseudo-time inference. This study highlights DB-AAE’s effectiveness and potential as a valuable tool for enhancing the quality and reliability of downstream analyses in scRNA-seq research.

## Highlights

• The dynamic batching adversarial autoencoder (DB-AAE) excels at denoising scRNA-seq datasets
• DB-AAE enhances resolution of pseudo-time inference
• DB-AAE outperforms other methods in denoising accuracy and biological signal preservation

## Graphical abstract

**Figure:**
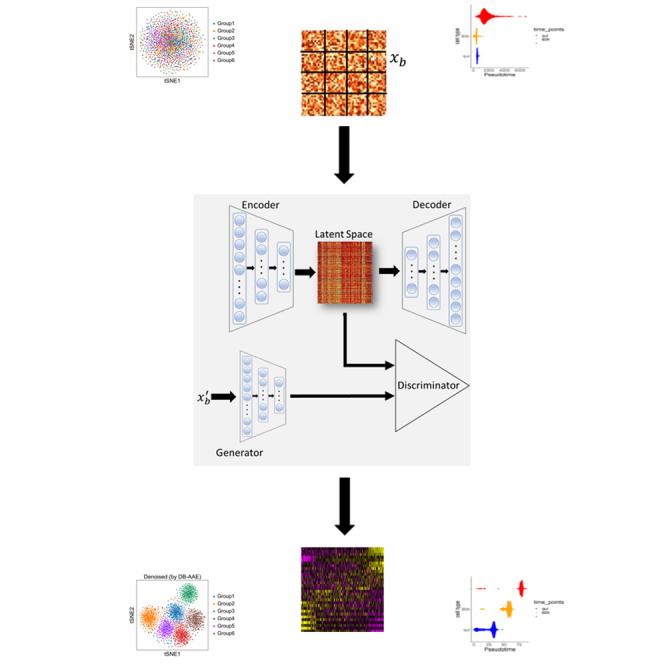

## Introduction

Single-cell RNA sequencing (scRNA-seq) has revolutionized gene expression profiling by revealing the transcriptome of individual cells. This method has provided valuable insights into cell heterogeneity, facilitated the discovery of rare cell populations, and enhanced our understanding of the molecular mechanisms underlying cellular function and disease.^1^ Despite important analytical advances, scRNA-seq still faces certain technical challenges, including low capture rates and dropout events. These limitations introduce noise that can interfere with data analysis and interpretation.

Dropout is a phenomenon observed when a given gene transcript is expressed at a low or moderate expression in one cell but is not detected in another cell of the same cell-type population.^2^ It occurs due to low sequencing depth, amplification bias, or biological factors, and can impact on downstream analysis such as clustering, trajectory analysis, and differential expression analysis. To mitigate the effects of dropout, numerous imputation or denoising methods have been developed that can be categorized into matrix factorization, nearest-neighbor method, probabilistic model, and deep learning-based method.^3^

Matrix factorization decomposes a matrix into lower-rank matrices to approximate the original matrix and estimates missing values based on data patterns.^4^ The accuracy of the imputed values depends on the characteristics of the data and the selection of factorization method and hyperparameters. Nearest-neighbor methods, such as K-nearest neighbors (KNN) imputation, estimate missing values by considering values from the nearest neighbors.^5^ KNN has a high computational cost to impute or denoise large datasets, and the accuracy decreases if the proportion of missing value is high in the dataset, or the missing values are not related to the key values. Probabilistic models, like the zero-inflated negative binomial (ZINB) model and Gaussian mixture model (GMM), infer missing values based on observed information and distribution assumptions.^3^ While useful, these methods can introduce biases in the denoised dataset and struggles to accurately denoise datasets, when the proportion of missing values is high, or the distribution of missing values is non-random.

Deep learning methods, specifically autoencoders, have been developed to capture non-linear relationships in scRNA-seq data.^6^^,^^7^^,^^8^^,^^9^ Autoencoders use feature extraction and latent space reconstruction to reduce noise and impute missing values. However, they can be sensitive to sparse data and batch effects.^10^ To address these issues, the deep count autoencoder (DCA) combines autoencoders with a negative binomial model to capture missing values and mitigate batch effects.^11^ Since mean and dispersion of an input matrix are used for the reconstruction of latent space, DCA can suffer overfitting and information loss. Variational autoencoders (VAEs) further improve upon this approach by incorporating a probabilistic generative framework, creating a smoother latent space, and capturing complex non-linear patterns among gene expression values.^12^ However, VAEs can suffer from mode collapse or loss of informative features in the latent space if lowly expressed genes in the dataset do not follow a particular statistical distribution such as negative binomial or Gaussian.

In this paper, our objective is to tackle the challenges associated with imputation and denoising in scRNA-seq data using a novel generative framework that leverages the power of adversarial autoencoders (AAEs). AAE combines autoencoders and generative adversarial networks (GANs).^13^ Traditional AAE frameworks primarily focus on training the generator and the encoder through the adversarial network to generate realistic outputs, making it difficult for the discriminator to distinguish between the generated and real data. Originally designed for synthesizing realistic images, AAEs with statistical models have been applied in scRNA-seq data for tasks such as dimension reduction, clustering, and integration.^14^^,^^15^ However, the potential of AAEs in denoising and imputing scRNA-seq datasets remains underexplored. In addition, while traditional AAEs exhibit good performance in the analysis of scRNA-seq data,^15^ there is a risk of information loss if the variance of gene expression does not follow the statistical models.^10^ To enhance denoising performance and mitigate information loss during analysis, we propose the dynamic batching adversarial autoencoder (DB-AAE) employing a competitive model that directly captures optimal features from input data rather than using statistical models. Batching is one of pivotal techniques in deep learning, wherein multiple input samples are processed concurrently as a batch.^16^ Numerous studies^17^^,^^18^^,^^19^^,^^20^ emphasize the crucial impact of batch size on the performance of training in deep neural networks. A large batch size may become stuck at local minima, while a small batch size can lead the loss function to converge to a biased minimum.^16^ Adapting the batch size according to the dataset’s characteristics has been shown to enhance the efficiency of neural network algorithms.^16^ To dynamically adjust the batch size during neural network training, three prominent algorithms are considered. First, random search^21^ involves the random selection of combinations of batch sizes. Second, Bayesian optimization^22^ utilizes Bayes Theorem to guide the search for the optimal batch size. Lastly, the Hyperband approach,^23^ a variant of random search, aims to determine the best resource allocation for adjusting the batching size. In our research, we employ the Hyperband algorithm for dynamic batching in AAEs to enhance the reconstruction performance and converge to an optimal minimum in the loss function. DB-AAE excels at retaining important features, such as cell type-specific gene expression patterns, even in the presence of noise in scRNA-seq data. This enhanced feature preservation significantly improves the reliability and accuracy of downstream analysis tasks such as clustering and pseudo-time inference.

We tested and performed a comprehensive evaluation of our proposed method, comparing it with other commonly used approaches, using both simulated and real datasets. Our analysis indicates that DB-AAE surpasses the performance of other methods in terms of denoising accuracy and preservation of biological signal. Moreover, our findings indicate that this method can significantly enhance the accuracy of other algorithms specifically designed for pseudo-time inference. These results not only validate the effectiveness of our approach but also emphasize its potential as a valuable tool for improving the quality and reliability of downstream analyses in scRNA-seq analysis.

## Results

### DB-AAE improves denoising of simulated scRNA-seq data

**Figure 1:**
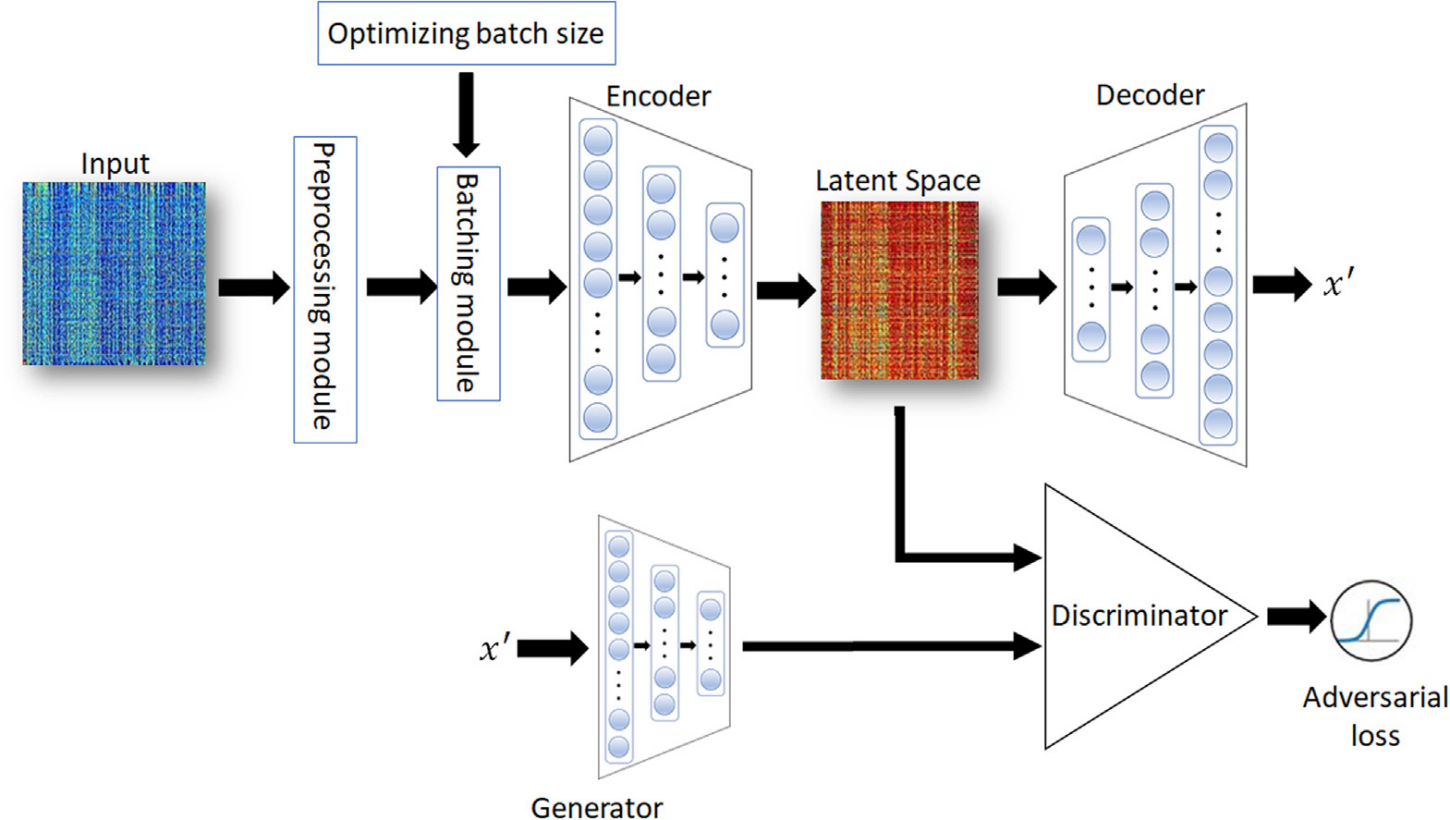
Dynamic batching adversarial autoencoder (DB-AAE) Schematic illustration of the different components of the DB-AAE. Blue circles represent nodes, $x^{\prime }$ represent reconstructed output in the DB-AAE structure. After preprocessing data, the encoder generates the authentic latent space using the current input batch, while the generator creates a simulated latent space by emulating the characteristics of the output through the autoencoder with the prior input batch during training. The encoder and decoder components are optimized until the discriminator cannot differentiate between the true and simulated latent spaces across the entire batch.

AAE is a deep neural network that combines the advantages of autoencoders and GANs to facilitate unsupervised learning tasks and generate new samples that closely resemble the input data by sampling from a learned latent space, and there are several advantages of AAEs compared to traditional GANs. First, since AAEs are designed to perform both generative and reconstructive tasks, AAEs can be efficient for tasks such as data denoising and imputation. Second, AAEs can control the generation process of latent space because they explicitly encode input data into a latent space. This makes it easier to decode output data with specific characteristics of the input. However, GANs do not provide a direct mapping from input to a latent space. AAE consists of three key components: encoder, decoder, and adversary modules (Figure 1). The encoder transforms input data into a lower-dimensional latent space, while a decoder reconstructs the input data from the latent space. The adversary modules encompass a generator and discriminator. The generator utilizes the latent space encoded by samples from the input data to produce synthetic data, whereas the discriminator distinguishes between the synthetic data and real data obtained from the original input’s latent space. In AAE, the encoder and decoder components are optimized in such a way that the discriminator cannot differentiate between synthetic samples generated by the generator and real data. This adversarial training process empowers the AAE to acquire a more meaningful and structured representation of the latent space.^13^ For their optimization, several statistical models have been used but information may be lost if the distribution does not follow statistical models.^10^ To remedy this potential loss, we implemented DB-AAE containing a novel adversarial framework with dynamic batching by sampled inputs.

**Figure 2:**
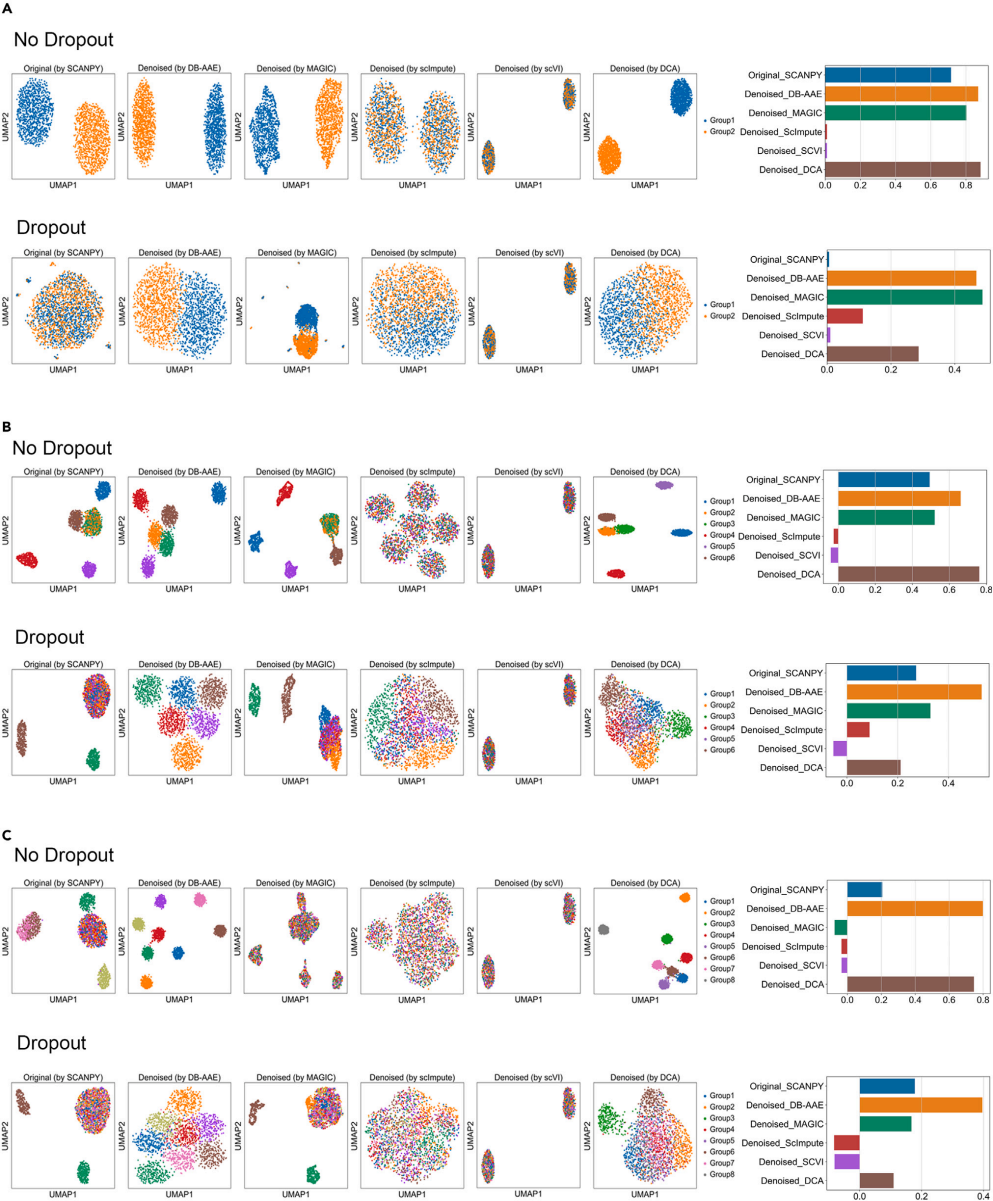
Identification of cell types in simulated data (A) UMAP (left) and silhouette score bar (right) plots of two distinct cell types without dropout noise (top) and with dropout noise (bottom) using different algorithms. (B) Six distinct virtual cell types without dropout noise (top) and with dropout noise (bottom). (C) Eight distinct virtual cell types without dropout noise (top) and with dropout noise (bottom).

To investigate the characteristics of the DB-AAE model, we conducted performance evaluation using simulated scRNA-seq data generated by the Splatter package^24^ after creating a count matrix consisting of 200 genes across 2,000 cells. We modified the simulation to introduce variations in the number of cell types (two, six, or eight virtual cell types) under either dropout or non-dropout conditions. Clustering efficacy and denoising capabilities of the DB-AAE model were evaluated by comparing it to five other methods, SCANPY, MAGIC (Markov affinity-based graph imputation of cells),^25^ DCA,^11^ scImupte,^26^ and SCVI (single cell variational inference).^12^ Figure 2 (left panels) illustrates the clustering results obtained by each method in the uniform manifold approximation and projection (UMAP) dimension. The performance of each clustering was assessed using the silhouette score (SC).^7^^,^^27^ The SC (Figure 2, right panels) quantifies the similarity of gene expression patterns within a cluster and the dissimilarity between different clusters, with values ranging from −1 to +1.^28^ An SC approaching 1 indicates that the clustering results are well-defined and that the cells are appropriately assigned to their respective clusters, suggesting a more reliable and meaningful clustering outcome. In the absence of dropout-induced noise in the small number of groups, DB-AAE, MAGIC, and DCA were able to regenerate clusters corresponding to the number of cell types, and their SCs did not differ significantly (no dropout in Figure 2A). However, after denoising datasets containing dropout noise in the large number of groups, DB-AAE exhibited superior performance compared to other algorithms in clustering cells belonging to the same cell types (dropout Figures 2B and 2C). In fact, while DB-AAE showed similar performance without noise in Figure 2 (no dropout), DB-AAE demonstrated superior performance compared to other methods in complex datasets with strong noise, as shown in Figure 2 (dropout), even though the silhouette score of the original simulated dataset in dropout is negative because the dataset is highly diverse and thus difficult to cluster. These simulated results provide evidence that DB-AAE outperforms other methods in terms of denoising and clustering efficiency.

### DB-AAE favorably compares to other approaches in denoising real scRNA-seq data

**Figure 3:**
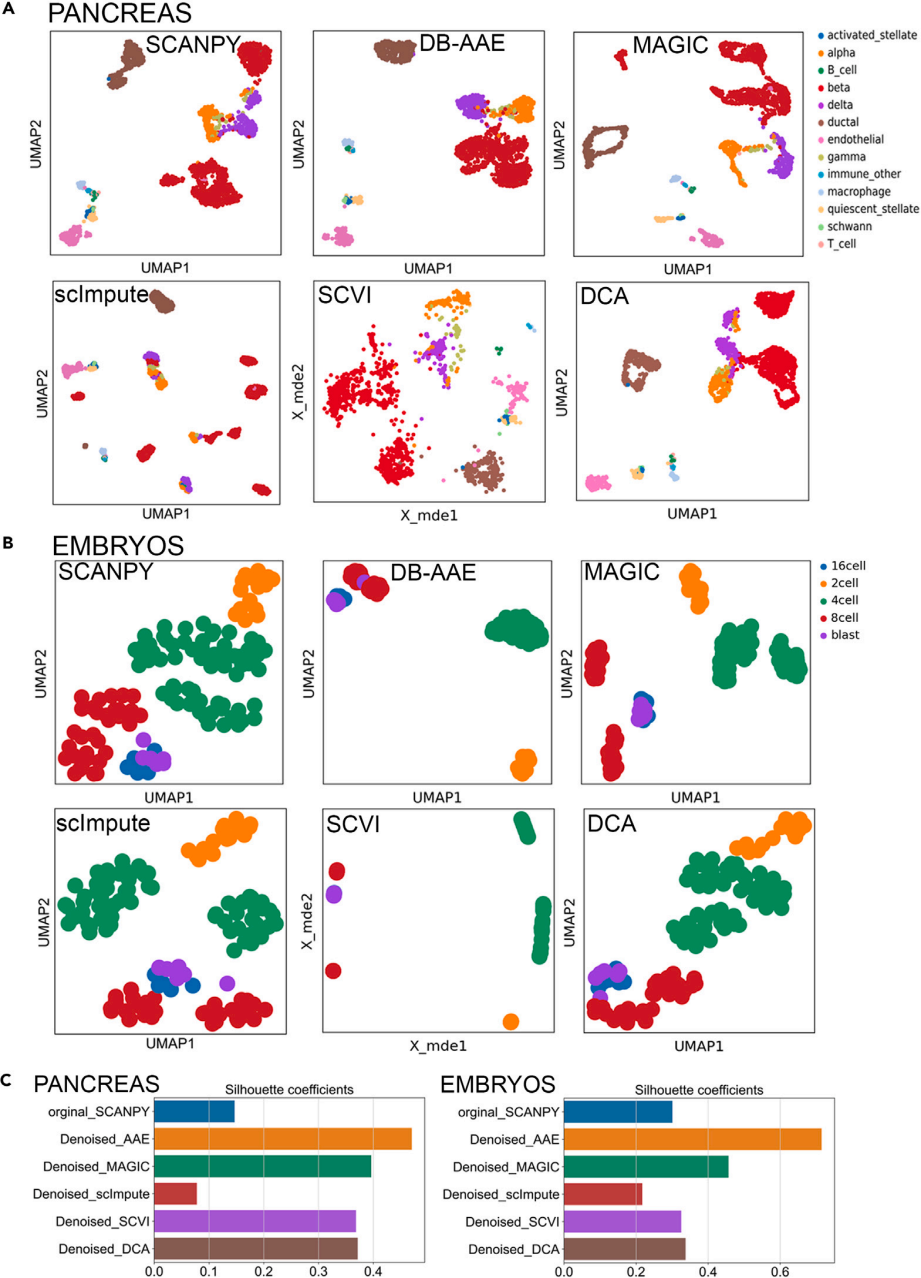
Performance of cell-based clustering with five different methods in biological data (A) UMAP plot of thirteen cell types from a pancreas dataset. (B) UMAP plot of five cell types from embryo dataset. (C) Silhouette score bar plots of thirteen cell types from a pancreas (left) and five cell types from embryo (right) dataset.

The denoising performance of DB-AAE was compared to five popular methods (SCANPY for clustering,^29^ MAGIC using Markov affinity-based graph imputation,^25^ DCA using deep count autoencoder,^11^ scImupte using a statistical method^26^ and SCVI using variational autoencoder^12^) using ten published scRNA-seq datasets reported in Table 1. Our aim was to assess DB-AAE’s ability to capture cell-based clusters in datasets with complex cell heterogeneity. In Figure 3A, we first present clustering results of a scRNA-seq mouse pancreas dataset.^30^ Compared to the original dataset (SCANPY Figure 3A), DB-AAE more effectively clustered endocrine pancreatic cells (alpha, beta, and delta cells) which were clearly distinct from exocrine ductal pancreatic cells (Figure 3A). Moreover, the similarity among cells belonging to the same cell type obtained by DB-AAE was improved compared to other methods. For instance, insulin-secreting pancreatic beta cells were assigned to one cluster by DB-AAE while other approaches assigned them to two or more clusters (Figure 3A, red clusters). We further analyzed the functional characteristics of the clusters related to pancreatic beta cells using gene ontology (GO) analysis.^31^ The three clusters identified as pancreatic beta cells in the original dataset (Figure S1A, left panel, labeled as c0, c1, and c2) shared GO terms not directly related to their endocrine function (Figure S1A, right panel). DB-AAE grouped these three clusters (Figure S1B, left panel) and identified “insulin receptor binding” term, related to pancreatic function,^32^ in each of the clusters c0, c1, and c2 (Figure S1B, right panel). Thus, DB-AAE denoising improved accuracy of GO analysis allowing identification of a critical function of pancreatic beta cells which was not evident in the original dataset. To assess the potential impact of biological overfitting on the analysis of a small-sized dataset, we analyzed scRNA-seq dataset comprising blastomeres of mouse embryos (124 cells), spanning zygote to late blastocyst stages.^33^ As shown in Figure 3B, DB-AAE successfully identified all clusters associated with cells of five developmental stages. Also in this case, as observed for the pancreas dataset, DB-AAE tightly assigned cells of the same developmental stage to a unique cluster (Figure 3B). Adjacent clusters identify cells with similar genetic-functional characteristics.^34^^,^^35^ Silhouette scores were calculated for the mouse pancreas dataset^30^ analyzed in Figure 3C (left panel) and embryo scRNA-seq dataset^33^ (Figure 3C, right panel). DB-AAE consistently achieved higher scores than the other methods across the two datasets. In both cases, but especially for the embryo datasets, DB-AAE generated clusters with a closer internal distance between cells compared to the other methods.

**Table 1:** References of datasets employed to evaluate performance and pseudo-time inference

Dataset	Tissue	# of cell	# of cell type	Accession ID	Reference
Clustering efficiency					
baron	Mouse pancreas	1886	13	GSE84133	Baron et al.^30^
zeisel	Mouse brain	3005	9	GSE60361	Zeisel et al.^36^
goolam	Mouse embryo	124	5	E-MTAB-3321	Goolam et al.^33^
xin	Human pancreas	1600	8	GSE81608	Xin et al.^37^
lake	Human brain	3042	16	phs000833.v3.p1	Lake et al. 2016
Slyper	Human blood	13316	8	SCP345	Tran et al.^38^
deng	Mouse embryo	268	6	GSE45719	Deng et al.^39^
Wang	Human pancreas	457	7	GSE83139	Wang et al.^40^
Muraro	Human pancreas	2126	10	GSE85241	Muraro et al.^41^
usoskin	Mouse brain	622	4	GSE59739	Usoskin et al.^42^
Pseudo time inference					
sartorelli	Mouse muscle	11046	3	GSE126834	Dell'Orso et al.^43^
ponce	Mouse pancreas	36351	4	GSE132188	Bastidas et al.^44^
treut	Mouse embryo	315	5	GSE67310	Treutlein et al.^45^
qiu	Mouse pancreas	575	7	GSE87375	Qiu et al.^46^
yuzwa	Mouse cortex	6000	4	GSE107122	Yuzwa et al.^47^
vlado	Mouse cerebellum	55000	8	GSE118068	Vladoiu et al.^48^

**Figure 4:**
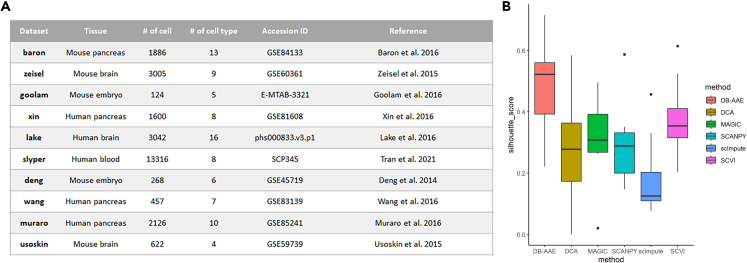
Evaluation of clustering performance using ten datasets with five different methods (A) Reference for the ten datasets employed for performance evaluation. (B) Comparison of average silhouette scores for the ten datasets obtained with different algorithms.

To conduct a thorough assessment of DB-AAE performance, we utilized and aggregated ten distinct datasets (Figure 4A), each containing various cell types, ranging from hundreds to thousands, derived from either human or mouse samples. The comparison of silhouette scores across these datasets provides a robust evaluation using six different methods. The average silhouette scores for each method using the ten datasets are presented in Figure 4B. Remarkably, DB-AAE consistently outperformed all other methods across all ten scRNA-seq datasets.

**Figure 5:**
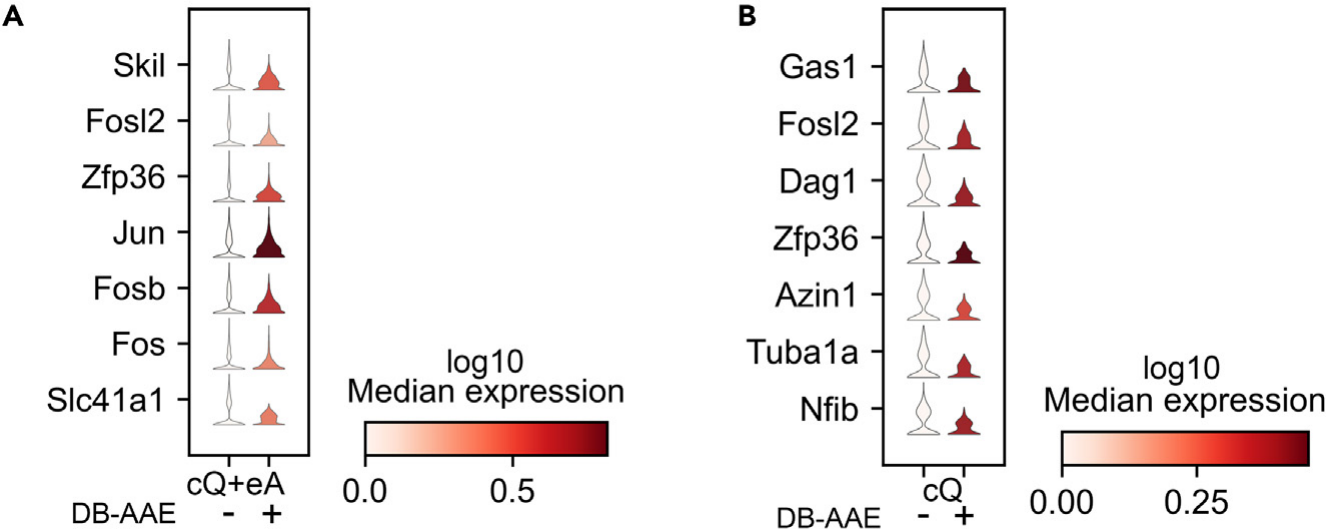
Impact of denoising by DB-AAE in gene-expressing patterns (A) Recovery of gene transcripts in close-to-quiescent and early activated (cQ + eA) MuSCs before and after denoising with DB-AAE. (B) Recovery of gene transcripts in cQ MuSCs before and after denoising with DB-AAE.

Next, we focused on scRNA-seq datasets derived from skeletal muscle stem cells (MuSCs) to check the biological impact of DB-AAE.^43^ In homeostatic condition, MuSCs are quiescent, have a low metabolic rate^49^ and a widespread low level of transcription,^50^ making it difficult to recover rare transcripts. Isolation procedures lead to transcriptional changes associated with MuSCs activation.^50^^,^^51^ We employed DB-AAE to assess its capability in detecting expression of rare transcripts expressed in FACS-isolated MuSCs consisting of close-to-quiescence (cQ) and early-activated (eA) MuSCs.^43^ Prior to employing DB-AAE, rare transcripts were barely detected and DB-AAE improved their identification (Figure 5A). Next, we wished to evaluate transcripts expressed in cQ MuSCs.^52^ Also in this case, DB-AAE greatly improved detection of lowly expressed transcripts (Figure 5B). These results highlight the capability of DB-AAE not only to remove noise but also to recover valuable gene expression patterns that might otherwise be missed.

### DB-AAE enhances resolution of pseudo-time inference

**Figure 6:**
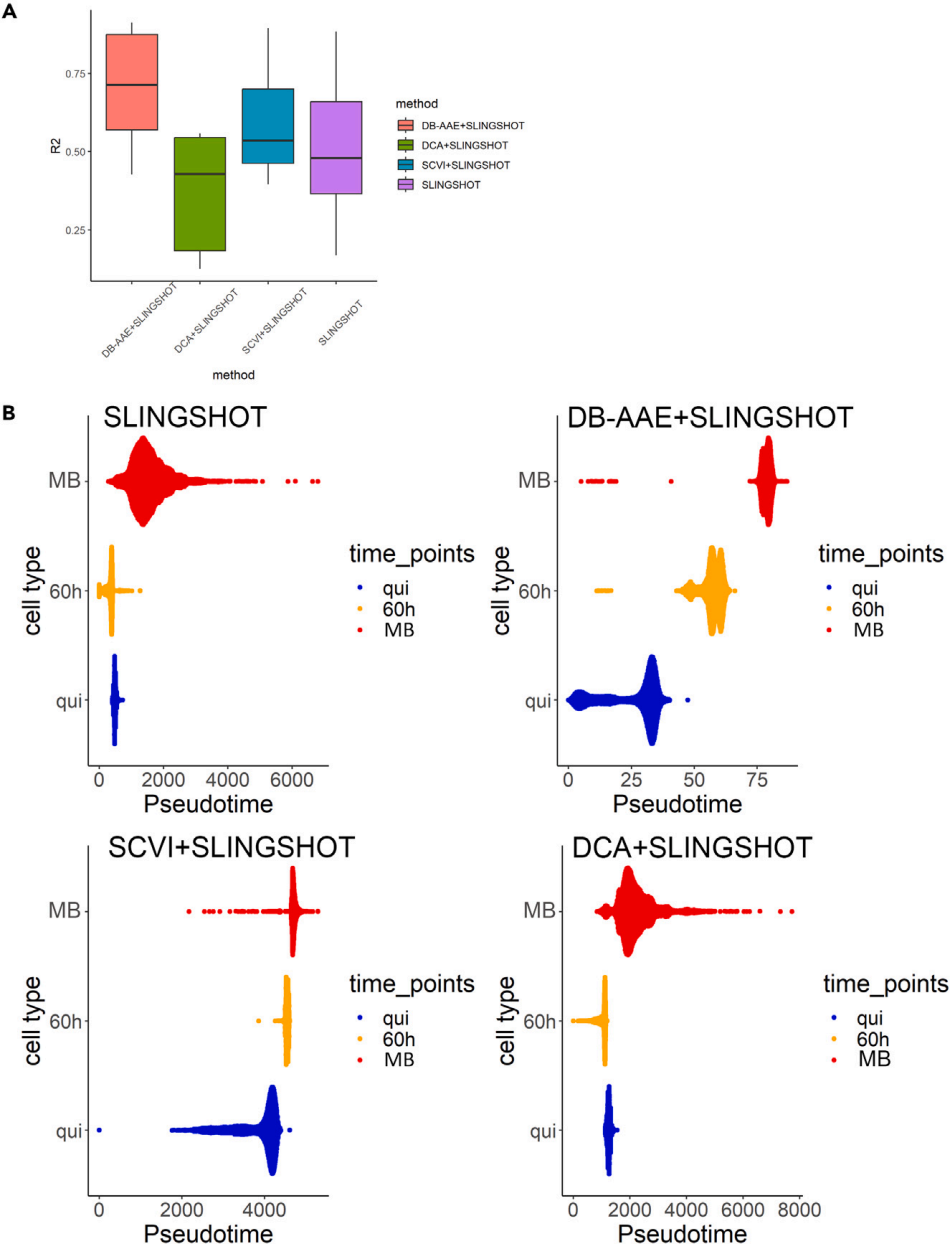
Impact of denoising by DB-AAE in pseudo-time inference (A) Comparison of average squared R scores for five datasets after processing with four different combined algorithms. (B) Pseudo-time ordering of homeostatic MuSCs (quiescent, qui), activated MuSCs (60 h after injury) and proliferating myoblasts (MB) after data processing with four different combined algorithms.

Pseudo-time inference is one of important procedures in the analysis of the single cell transcriptome and computationally infers the order of these cells along developmental trajectories.^53^ Even though DB-AAE does not have a function of pseudo-time inference, it can support to improve the performance of other algorithms for the inference. Therefore, we performed an evaluation of pseudo-time inference after applying denoising techniques to determine the impact of denoising DB-AAE on pseudo-time inference. We conducted a comprehensive evaluation of three popular autoencoder approaches, aiming to assess their performance on six diverse datasets (Table 1, pseudo-time inference). These datasets encompass various developmental cell states ranging from three to eight, originating from different tissues and organs. After utilizing denoising techniques, we inferred pseudo-time using the widely used Slingshot algorithm.^54^ Next, we employed squared R scores to measure the correlation between the inferred pseudo time and annotated developmental stages.^38^^,^^55^ A higher squared R score indicates a closer alignment between the predicted pseudo-time and the annotated stages. In this analysis, DB-AAE consistently outperformed the other methods across all scRNA-seq datasets (Figure 6A). In Figure 6B, we present the results obtained for MuSCs datasets,^43^ comprising three differentiation stages (quiescent MuSCs, activated MuSCs isolated 60 h after muscle injury, and culture myoblasts). Our analysis revealed that, by combining DB-AAE denoised with Slingshot, we achieved higher accurate predictions of the pseudo-time corresponding to the three differentiation cell stages, compared to other methods. These findings highlight the superiority of the DB-AAE denoising method in combination with Slingshot for pseudo-time inference and accurate prediction of developmental stages.

## Discussion

scRNA-seq provides valuable insights into the diversity of cells and the mechanisms underlying diseases.^1^ Nonetheless, this approach comes with challenges, including issues such as limited capture rates and dropout events, which have the potential to introduce undesired variability in the process of data analysis.^2^ Even though numerous imputations or denoising methods have been developed to mitigate the effects of the issues, there are still technical limitations.^3^

In this study, we introduce a novel generative framework called DB-AAE to address the challenges associated with denoising and imputation in scRNA-seq data. This framework leverages the power of AAEs, which combine autoencoders and GANs. While traditional AAEs rely on statistical modeling to generate a latent space that captures expression patterns within scRNA-seq data, the DB-AAE introduces a paradigm shift by employing an adversarial technique. This technique directly samples from the input data to create the latent space, circumventing the limitations of statistical modeling. To evaluate the effectiveness of DB-AAE, we conducted comprehensive testing using both simulated and real datasets. The proposed method was compared to other commonly used approaches such as MAGIC,^25^ DCA,^11^ scImupte^26^ and SCVI,^12^ and the analysis demonstrated that DB-AAE outperformed other methods in terms of denoising accuracy and the preservation of biological signal. Additionally, the results showed that DB-AAE significantly improved the accuracy of other algorithms designed for pseudo-time inference, including Slingshot. These findings not only validate the effectiveness of the proposed approach but also highlight its potential as a valuable tool for enhancing the quality and reliability of downstream analyses in scRNA-seq research.

Throughout this study, we show that generative adversarial methods based on deep learning neural networks provide a promising alternative to existing methods. This approach also preserves important features such as cell type-specific gene expression patterns and robustness to noise in scRNA-seq data. DB-AAE can improve the reliability of downstream analyses such as clustering and pseudo-time inference by minimizing information loss during analysis. The DB-AAE framework can be integrated with other existing single-cell sequencing analysis methods to create more comprehensive pipelines. For example, combining DB-AAE with existing clustering algorithms, dimensionality reduction techniques, or trajectory inference methods could lead to more robust and accurate downstream analyses. In addition, optimization techniques can be explored to enhance the training process and convergence of the DB-AAE framework. Techniques like advanced regularization methods, different loss functions, or learning rate can be investigated to improve the stability and efficiency of the model. Additionally, incorporating techniques such as pre-training on related datasets could be explored to leverage prior knowledge and improve performance on specific datasets.

### Limitations of the study

Since DB-AAE are implemented on a deep-learning model, our study is limited to provide more detailed insights into the precise acquisition and utilization of specific features or gene expression patterns by the model. This is due to the intricate nature of deep learning models, composed of numerous layers with complex interactions between nodes. As a result, internal workings or processes are not easily understandable or interpretable. Although the DB-AAE framework has demonstrated effectiveness on both simulated and real datasets used in the study, further evaluation of additional datasets from different tissues, organisms, or experimental conditions is required. In addition, since the performance of the DB-AAE framework is sensitive to hyperparameters,^56^ a comprehensive hyperparameter tuning would be necessary to ensure the stability and robustness of the method.

## STAR★Methods

### Key resources table

**Table:** 

REAGENT or RESOURCE	SOURCE	IDENTIFIER
**Deposited data**		

Code for development and evaluation	This paper	https://github.com/LMSCGR/DB-AAE

**Software and algorithms**		

Scanpy	Wolf et al.^29^	https://github.com/scverse/scanpy
MAGIC	van Dijk et al.^25^	https://github.com/pkathail/magic
DCA	Eraslan et al.^11^	https://github.com/theislab/dca
scImpute	Li and Li,^57^	https://github.com/Vivianstats/scImpute
SCVI	Lopez et al.^12^	https://github.com/scverse/scvi-tools
Slingshot	Street et al.^54^	https://github.com/kstreet13/slingshot
R	The R Project for Statistical Computing	https://www.r-project.org/
Python	Python Software Foundation	https://www.python.org/downloads/source/
Custom scripts	This paper	https://doi.org/10.5281/zenodo.10478925

### Resource availability

#### Lead contact

Further information and requests for resources should be directed and will be fulfilled by the lead contact, Vittorio Sartorelli (vittorio.sartorelli@nih.gov).

#### Materials availability

This study did not generate new unique reagents.

#### Data and code availability

This paper analyzes existing publicly available data. The accession numbers of the datasets employed in this study are listed in Table 1.

All original codes have been deposited at GitHub (https://github.com/LMSCGR/DB-AAE) and are publicly available as of the date of publication.

Any additional information required to reanalyze the data reported in this paper is available from the lead contact upon request.

### Method details

#### Data preprocessing

Table 1 describes 16 single-cell datasets (clustering efficiency:10 and pseudo time inference:6) used in data analysis. Some datasets^30^^,^^36^^,^^37^^,^^39^^,^^40^^,^^41^^,^^42^^,^^43^^,^^44^^,^^45^^,^^46^^,^^47^^,^^48^ were downloaded from Gene Expression Omnibus (GEO). Other datasets^33^^,^^38^ were downloaded from Broad Institute Single Cell Portal. After removing cells with ambiguous labels in the datasets, we converted the datasets into the standard h5 anndata format for training DB-AAE and evaluation of the performance.

#### Dynamic batching adversarial autoencoder

We designed a modified version of AAE (AAE) to mitigate losing information during training procedure. Traditional AAE consists of three key components: encoder, decoder, and adversary modules (Figure 1). The encoder usually transforms input data into a lower-dimensional latent space, and the input of the encoder is normalized gene expression profile using highly variable genes annotated by dispersion-based method.^58^ First, after transforming the gene expressions to z-scores using Equation 1, we calculated the normalized variance of each gene and ranked the genes by the normalized variances. Finally, we selected genes as the input of encoder with high variances using preprocessing module in Figure 1.

(Equation 1) $$z_{mn}=\frac{G_{mn}-{\tilde{G}}_{m}}{\sigma_{m}}$$

where $z_{mn}$ is *Z* score of gene *m* in cell *n*, $G_{mn}$ is expressing value of gene *m* in cell *n*, ${\tilde{G}}_{m}$ is mean expressing value of gene *m*, $\sigma_{m}$ is the expected standard deviation of feature m derived from the global mean-variance.

Subsequently, we implemented dynamic batching procedures utilizing the Hyperband algorithm.^23^ As illustrated in Figure 1, we initialize the starting, ending, and increment values for the batching size within the optimizing batch size module. In each cycle, the input data are segmented into batches using predetermined step-ups from the optimizing module. The encoder generates the authentic latent space using the current input batch, while the generator creates a simulated latent space by emulating the characteristics of the output through the autoencoder with the prior input batch. The training process involves continuous iterations, with the autoencoder and discriminator refining their models until the discriminator can no longer differentiate between the true and simulated latent spaces across the entire batch.

Defining a selected gene expressing profile of cell m as input *x*, the architecture of AAE can be formulated as follows:

$$x_{b}=S\left(x\right)$$

$$z_{b}=l_{e}\left(x_{b}\right)$$

(Equation 2) $$a_{loss}=D\left(z_{b},n_{r}\right)$$

$$x_{b}^{\prime }=l_{d}\left(z_{b}\right)$$

$$x^{\prime }=\overset{n}{\underset{i=0}{⋃}}x_{bi}^{\prime }$$

where $x_{b}$ is an input batched from input data *x*, $z_{b}$ is the latent representation from the batched input $x_{b}$, $n_{r}$ is the latent representation from $x_{b}^{\prime }$, $x_{b}^{\prime }$ is reconstructed input from $x_{b}$, $a_{loss}$ is adversarial loss, S is batch sampling function, *l*_*e*_ is encoder layer, D is discriminator layer, *l*_*d*_ is decoder layer, U is union of $x_{b}^{\prime }$ , $x^{\prime }$ is reconstructed output from batched inputs*,* n is the number of batches.

The encoder layer is defined below:

(Equation 3) $$l_{e}=\text{LeakReLU}\left(XW_{e}\right)$$

where *X* represents input, *W*_*e*_ represents weight values in encoder layer.

The decoder layer is defined below:

(Equation 4) $$l_{d}=\text{LeakReLU}\left(ZW_{d}\right)$$

where *Z* represents latent matrix, *W*_*d*_ represents weight values in the decoder layer.

To complete adversarial training, DB-AAE uses a discriminator network to distinguish between the true latent space using the current input batch and a synthetic latent space by mimicking the features of the output generated in the preceding input batch to minimize reconstruction (autoencoder) and generator loss, while maximizing the discriminator loss.

For reconstruction loss, we used binary cross-entropy between batched input *x* and reconstructed output *x'* below:

(Equation 5) $$L_{rec}=-\frac{1}{N}\sum_{i=1}^{N}(x_{i}\;\log\left(x_{i}^{\prime }\right)+\left(1-x_{i}\right)\log\left({1-x}_{i}^{\prime }\right))$$

The generator loss is defined below:

(Equation 6) $$L_{gen}=-\frac{1}{N}\sum_{i=1}^{N}\log\left(1-D(l_{e}(x_{i}^{\prime }\right)))$$

The discriminator loss is defined below:

(Equation 7) $$L_{disc}=-\frac{1}{N}\sum_{i=1}^{N}\left(\log\left(D(l_{e}(x_{i}\right)\right)+\log\left(1-D(l_{e}(x_{i}^{\prime }\right)))$$

where N is batch size, D(x) represents the output of the discriminator.

Through these formulas, the autoencoder, generator, and discriminator are updated iteratively until DB-AAE discovers a balance between the reconstruction capability and the ability to generate realistic encoded samples.

After each cycle, the Batching Module stores the current batch size, accuracy, and the minimum loss function values of the autoencoder. A new batch size is then initialized for the subsequent cycle. This process is repeated until the batch size reaches its maximum value in the predetermined step-ups. The batch size associated with high accuracy and low minimum loss is selected from the results of all cycles. Using the chosen batch size, the DB-AAE performs a final training cycle to construct an optimal denoising model.

#### Hyperparameters

The encoder network dimensions are set to input-1024-512-512, where input stands for the dimension of input data, and the decoder has a symmetric structure with the encoder. In addition, the discriminator network is built with dimensions 512-256-1. the activation function of the last layer of encoder, decoder, and discriminator is relu, while fully connected layers are all activated by LeakyReLU. In the training stage, we utilize the optimizer RMSprop with learning rate 0.00002 for all the datasets.

#### Measurement of performance with other algorithms

Software and algorithms used for to evaluate the performance of DB-AAE are cited in the appropriate sections in the STAR methods. For the evaluation, we used silhouette score and *r* (unstandardized Pearson’s correlation).^2^ Silhouette score for clustering performance is calculated using the mean intra-cluster distance (a) and the mean nearest-cluster distance (b) for each sample, and the formula is defined below:

(Equation 8) $$s_{i}=\frac{b_{i}-a_{i}}{\max\left(b_{i},a_{i}\right)}$$,

*b*_*i*_ is the inter cluster distance defined as the average distance to closest cluster of data point I except for that it’s a part of

(Equation 9) $$b_{i}=\min_{k\neq i}\frac{1}{\left|C_{k}\right|}\sum_{j∊C_{k}}d\left(i,j\right)$$,

and *a*_*i*_ is the intra cluster distance defined as the average distance to all other points in the cluster to which it’s a part of

(Equation 10) $$a_{i}=\frac{1}{\left|c_{i}\right|-1}\sum_{j∊c_{i},i\neq j}d\left(i,j\right)$$

The value of silhouette score is between −1 and 1, and the value close to 1 means the clusters are well-defined and well-separated from each other. In addition, it shows that the data points within each cluster are more similar to each other than to points in other clusters.

*r*^*2*^ for the accuracy of pseudo time inference is calculated using Fit-regression model defined below:

(Equation 11) $$\hat{y}=a+bx,b=r\left(\frac{s_{y}}{s_{x}}\right)and\;a=\bar{y}-b\left(\bar{x}\right)$$

where $s_{y}$, $s_{x}$ is standard deviations of yandx, $\bar{y}$, $\bar{x}$ are means of yandx, r is unstandardized Pearson’s correlation. The high value of *r*^*2*^ indicates that actual predicted pseudo times are close to target or reference timepoints.
