# Supplementary material for: A deep learning adversarial autoencoder with dynamic batching displays high performance in denoising and ordering scRNA-seq data
Source: iScience. 2024 Jan 30;27(3):109027. doi: 10.1016/j.isci.2024.109027 (PMC10867661; doi:10.1016/j.isci.2024.109027)
Supplement: Document S1. Figure S1 [file mmc1.pdf]

iScience, Volume *27*

Supplemental information

A deep learning adversarial autoencoder with dynamic batching displays high performance in denoising and ordering scRNA-seq data

Kyung Dae Ko and Vittorio Sartorelli

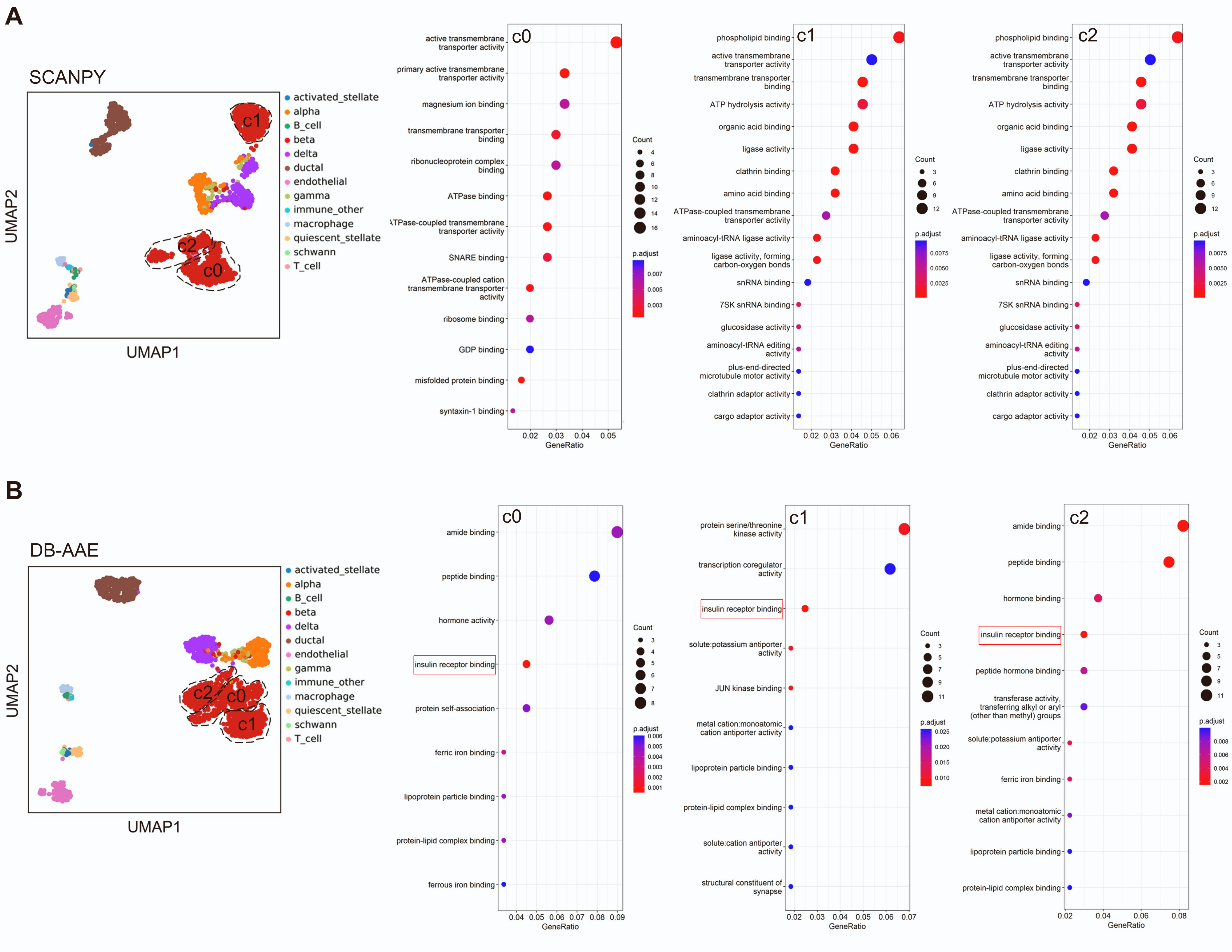
A
SCANPY
UMAP2
UMAP1
c0
c1
c2
activated_stellate
alpha
B_cell
beta
delta
ductal
endothelial
gamma
immune_other
macrophage
quiescent_stellate
schwann
T_cell
active transmembrane transporter activity
primary active transmembrane transporter activity
magnesium ion binding
transmembrane transporter binding
ribonucleoprotein complex binding
ATPase binding
ATPase-coupled transmembrane transporter activity
SNARE binding
ATPase-coupled cation transmembrane transporter activity
ribosome binding
GDP binding
misfolded protein binding
syntaxin-1 binding
GeneRatio
Count
p.adjust
phospholipid binding
ATP hydrolysis activity
organic acid binding
ligase activity
clathrin binding
amino acid binding
aminoacyl-tRNA ligase activity
ligase activity, forming carbon-oxygen bonds
snRNA binding
7SK snRNA binding
glucosidase activity
aminoacyl-tRNA editing activity
plus-end-directed microtubule motor activity
clathrin adaptor activity
cargo adaptor activity
B
DB-AAE
amide binding
peptide binding
hormone activity
insulin receptor binding
protein self-association
ferric iron binding
lipoprotein particle binding
protein-lipid complex binding
ferrous iron binding
protein serine/threonine kinase activity
transcription coregulator activity
solute:potassium antiporter activity
JUN kinase binding
metal cation:monoatomic cation antiporter activity
solute:cation antiporter activity
structural constituent of synapse
hormone binding
peptide hormone binding
transferase activity, transferring alkyl or aryl (other than methyl) groups

Supplemental Figures

Supplemental Figure S1 (related to Figure 3)

(A) UMAP plot of thirteen cell types from the pancreas dataset reported in Figure 3 (left panel). Beta cells are identified in cluster 0, 1, 2 (c0, c1, c2). Gene ontology of the individual beta cells clusters is presented on the right panel. (B) UMAP plot of thirteen cell types from the pancreas dataset after DB-AAE denoising (left panel). Beta cells are identified as cluster 0, 1, 2 (c0, c1, c2). Gene ontology of the individual beta cells clusters is presented on the right panel.
